# Exploratory Laparotomy With Enhanced Recovery Under Combined Thoracic Segmental Spinal and Epidural Anesthesia in a High-Risk Elderly Patient: A Case Report

**DOI:** 10.7759/cureus.90709

**Published:** 2025-08-21

**Authors:** Abhirami Ravindran, Imran Ahmed Khan, Sanjeev Shetty

**Affiliations:** 1 Anesthesiology, Bangalore Hospital Kengeri, Bangalore, IND; 2 Community Medicine, KMC Medical College and Hospital, Maharaj Ganj, IND; 3 Surgery, Bangalore Hospital Kengeri, Bangalore, IND

**Keywords:** epidural anesthesia, incisional hernia, obesity, segmental thoracic spinal anesthesia, small bowel obstruction, starvation ketosis

## Abstract

Incisional hernia after an abdominal surgery may be complicated by small bowel obstruction (SBO), which needs prompt surgical intervention, commonly under general anesthesia (GA). Elderly patients with comorbidities are at high risk for anesthesia. Thoracic segmental spinal anesthesia (TSSA) is a feasible alternative in such high-risk cases where extended anesthesia duration may be achieved by combining epidural. We report the case of a 66-year-old obese female patient presenting with a three-day history of SBO with starvation ketosis and bilateral pleural effusion who underwent exploratory laparotomy under TSSA at T10 combined with epidural anesthesia at T11. The 4-hour procedure, involving 500 mL blood loss replaced with one unit of packed red blood cells, was successfully completed with stable vitals. The hypotension following TSSA responded to a bolus of 6 mg of ephedrine, and low-dose noradrenaline infusion was used to counteract further hemodynamic instability. The patient remained pain-free postoperatively, and noradrenaline was discontinued. Postoperative ketone levels normalized with early mobilization and enhanced recovery. This case report emphasizes that combined thoracic segmental spinal and epidural anesthesia can be used safely in managing complex high-risk surgical cases with a tailored anesthetic technique. Further studies with large samples are required to validate these findings.

## Introduction

Prior abdominal surgery may lead to adhesions and small bowel obstruction (SBO), often associated with incisional hernia. Exploratory laparotomy under general anesthesia (GA) is commonly performed when conservative treatment fails. Comorbidities, including obesity, hypertension, etc., increase perioperative risks, necessitating careful anesthetic management [1]. The prevalence of obesity is rising rapidly worldwide and is now recognized as a critical public health challenge. A recent projection estimates that nearly 1 in 5 adults globally are living with obesity, with rates predicted to reach 18% in men and 21% in women by the year 2025. [2] Starvation ketosis, resulting from prolonged fasting, leads to metabolic abnormalities and electrolyte imbalance, potentially exacerbating hemodynamic instability and propensity for delayed recovery [3]. Regional anesthesia techniques, such as spinal and epidural anesthesia, offer advantages in high-risk patients by minimizing postoperative complications and sedation requirements [4]. Regional anesthesia also avoids complications related to airway instrumentation, particularly in obese patients [5]. Thoracic segmental spinal anesthesia (TSSA), targeting specific dermatomes, combined with epidural (CSE), provides effective analgesia and anesthesia while preserving patient consciousness and ensuring early recovery [6,7]. This case report describes the successful use of CSE anesthesia for exploratory laparotomy in an obese patient with SBO, incisional hernia, bilateral pleural effusion, and starvation ketosis, highlighting its feasibility and benefits in complex surgical scenarios. This case has been reported along with the Surgical CAse REport (SCARE) guidelines [8].

## Case presentation

A 66-year-old female patient, weighing 120 kg (body mass index, 42 kg/m²), presented to the emergency department with a three-day history of abdominal pain and inability to pass stool and flatus.

The diagnostic workup clarifies an incisional hernia leading to SBO. Her medical history included hypertension, controlled with oral antihypertensives (Tab Amlodipine 5 mg once daily and Tab Telmisartan 40 mg at night). She was diagnosed with starvation ketosis secondary to prolonged fasting. The patient was started on intravenous fluids, including thiamine, dextrose, and saline. Preoperative evaluation revealed sinus tachycardia (130 bpm), a blood pressure of 110/70 mmHg, a normal electrocardiogram, and an echocardiogram. A chest X-ray revealed bilateral mild pleural effusion with blunting of costophrenic angles. Her blood investigation was within normal limits, including electrolyte levels, except for raised ketones (Table 1). Preoperative arterial blood gas (ABG) showed mild metabolic acidosis consistent with starvation ketosis (pH 7.31, pCO₂ 32 mmHg, HCO₃ 16 mmol/L, base excess -8 mEq/L).

**Table 1:** Preoperative laboratory results

Parameter	Result	Normal Range
Hemoglobin	12.8 g/dL	12.0–16.0 g/dL (female)
White Blood Count	8.2 × 10⁹/L	4.0–11.0 × 10⁹/L
Platelets	280 × 10⁹/L	150–400 × 10⁹/L
Sodium	138 mmol/L	135–145 mmol/L
Potassium	4.0 mmol/L	3.5–5.0 mmol/L
Creatinine	70 µmol/L	45–90 µmol/L (female)
Blood Glucose	5.5 mmol/L	4.0–7.8 mmol/L
Serum Ketones	1.8 mmol/L	< 0.6 mmol/L
Serum Lactate	1.2 mmol/L	0.5–2.2 mmol/L

A multidisciplinary team comprising an anesthesiologist, physician, surgeon, and intensivist discussed the case, and the patient was scheduled for an urgent exploratory laparotomy under CSE to relieve obstruction and incisional hernia repair.

**Figure 1:**
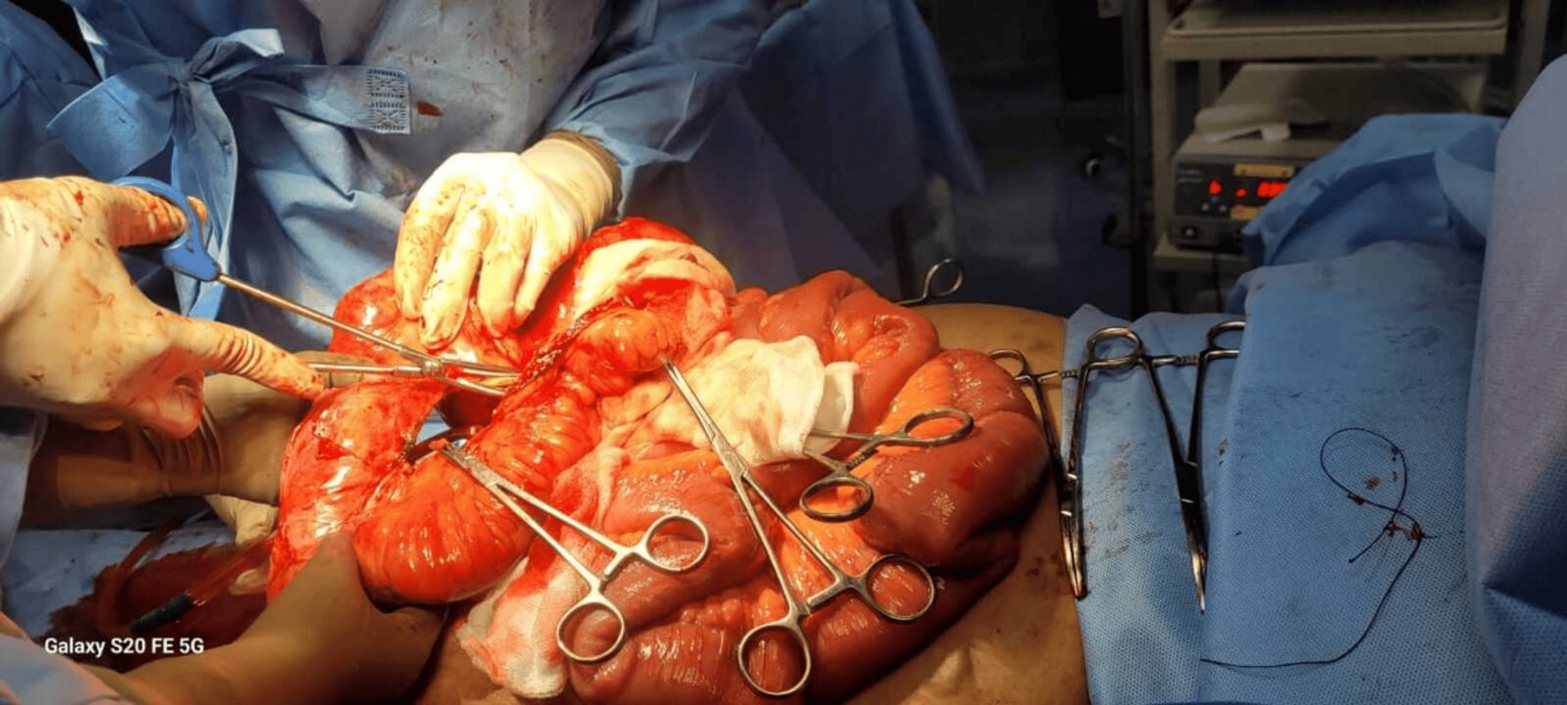
Intraoperative picture of patient showing incarcerated incisional hernia causing small bowel obstruction

A thorough preanesthetic checkup (PAC) was carried out, including airway examinations and assessment of possible difficulty due to obesity and other concerns. There was no history of obstructive sleep apnea. The neck was short with normal movements, and the thyromental distance was short. Mallampati score was 2. After obtaining informed consent for the procedure, the patient was shifted to the operating theater. Anticipating a difficult airway, we had a difficult airway cart kept ready with a video laryngoscope and a fibre optic on standby. Monitors were attached, and their functions were verified. Invasive lines (arterial and central venous) were placed under local anesthesia to closely monitor hemodynamics. An epidural catheter was inserted at T11 for intraoperative supplement and postoperative analgesia. TSSA was administered at the T10 level using 2.2 mL of hyperbaric bupivacaine (0.5%) with 5 μg of dexmedetomidine. There was a drop in BP to 80/60 mmHg three minutes after TSSA, which responded promptly to fluid and a 6 mg bolus of ephedrine. An exploratory laparotomy commenced, revealing an incarcerated incisional hernia causing SBO (Figure 1). The midline vertical incision spanned dermatomes T6 to L1. TSSA at T10 provided a sensory block from T4 to L2, with epidural supplementation ensuring coverage for the full incision site and prolonged duration. The procedure, lasting 4 hours, involved adhesiolysis, small bowel decompression, and hernia repair. Total blood loss was about 500 mL, which was replaced with one unit of packed red blood cells (PRBC).

For the first two hours, TSSA provided adequate surgical anesthesia. Subsequently, the epidural was activated with a five mL bolus of 0.5% bupivacaine and then a continuous infusion at a rate of six mL/hour for the rest of the intraoperative period. A 0.125% bupivacaine with two micrograms per mL of fentanyl was provided at 6 mL/hour for postoperative analgesia. A low dose of noradrenaline (1-2 mL/hour, approximately 4-8 μg/min) was started intraoperatively to maintain hemodynamic stability. No intraoperative sedation was used. Postoperatively, vitals stabilized (BP 124/78 mmHg, HR 90 bpm), with no further need for noradrenaline, which was discontinued. Postoperative ABG was normal (pH 7.38, PaCO₂ 39 mmHg, PaO₂ 92 mmHg, HCO₃ 24 mmol/L, and lactate 0.9 mmol/L). The epidural infusion continued for the next 48 hours for postoperative multimodal analgesia along with the injection of paracetamol 1 gm every eight hours. The patient sat up at four hours postoperative, ambulated at six hours postoperative, and had full independent mobility by day one. The patient was discharged on postoperative day five, with follow-up scheduled for two weeks, and was reported to have made a full recovery. The patient was satisfied with the perioperative course. Written informed consent was obtained for the publication of this case, with assurance to take stringent measures to conceal her identity.

## Discussion

This case demonstrates the successful use of CSE anesthesia with enhanced recovery for exploratory laparotomy in a high-risk obese patient with SBO and starvation ketosis. Obesity (BMI >30 kg/m²) presents with unique perioperative concerns, including difficult airway, hemodynamic instability, and postoperative pulmonary complications [9]. Starvation ketosis, characterized by elevated ketone levels due to prolonged fasting, can exacerbate metabolic stress and hypotension, particularly under anesthesia [3]. An obese patient with bilateral effusion, if given GA, would have been difficult to wean [10]. The choice of regional anesthesia over GA was made to avoid airway manipulation and postoperative pulmonary complications in view of obesity and pleural effusion. Decisions should be individualized, weighing patient comorbidities, procedure type, urgency, and institutional expertise. Table 2 provides a comparison between CSE and GA.

**Table 2:** Comparison of combined spinal epidural with general anesthesia GA: general anesthesia; NSAIDs: non-steroidal anti-inflammatory drugs; PONV: postoperative nausea vomiting

Management concerns	Combined Spinal-Epidural	General anesthesia
Intraoperative Considerations		
Airway Risks	Avoided (unless converted to GA)	Present (intubation, aspiration risk)
Hemodynamic Effects	Sympathetic blockade may cause hypotension, easily managed with fluids/vasopressors	Anesthesia/vasodilators may cause hypotension and tachycardia; airway manipulation can evoke stress responses
Early Pain Control	Excellent; dense analgesia perioperatively	Variable; requires multimodal analgesia with systemic and regional adjuncts
Muscle Relaxation	Lower limb and abdominal wall relaxation: variable; may need supplemental agents	Profound muscle relaxation (with neuromuscular blockers)
Postoperative Recovery		
Pain Control	Excellent (continuous epidural infusions)	Systemic analgesics are needed (opioids/NSAIDs); higher pain scores are common
Nausea and Vomiting	Lower incidence	Higher risk (especially with opioids and other agents used during GA)
Recovery Profile	Early bowel movement, less sedation, and earlier ambulation	Recovery is often within 1-2 hours for short procedures but delayed in high-risk or prolonged cases
Pulmonary Complications	Lower risk (no airway instrumentation, better cough reflex)	Lower pulmonary risk with modern techniques, but higher than pure regional in obese patients on prolonged ventilation

Starvation ketosis was managed with dextrose and saline infusion. Thiamine was added to prevent Wernicke's encephalopathy. CSE was found to be advantageous over GA for laparoscopic cholecystectomy [11]. Similarly, ipsilateral nephrectomy and hemicolectomy in a one-lung patient were conducted under CSE following an enhanced recovery after surgery protocol [12]. TSSA at the T10 interspinous level provided effective surgical anesthesia for the required surgical field, with dexmedetomidine enhancing the duration and quality of analgesia [13]. TSSA is safe if fine details are taken care of [14]. The epidural at T11 allowed for prolonged intraoperative and postoperative pain control, reducing opioid requirements and their associated risks, particularly in elderly people with obesity [15]. TSSA at T10 provided a segmental blockade for the midline incision (dermatomes T6-L1), which was suitable for this procedure. Hernia repair in an elderly patient with multiple comorbidities was safely conducted under CSE [16]. The observed initial hypotension is a known complication of SA, attributed to sympathetic blockade, but it was promptly managed in this case with a fluid bolus and a single dose of ephedrine, resulting in no adverse sequelae. Requiring noradrenaline support highlights the chances of hypotension during TSSA, though it was controlled without complications in this case. A recent meta-analysis reported a higher incidence of hypotension with TSSA in upper abdominal and breast surgeries. The same study also demonstrated several other benefits, such as superior pain control, reduced opioid requirements, shorter post-anesthesia care unit (PACU) stays, and reduced odds of postoperative nausea and vomiting (PONV) [17]. This supports TSSA's safety profile in high-risk patients. The propensity for large fluid shifts during prolonged exploratory laparotomy and initial dehydration necessitates invasive hemodynamic monitoring in such cases.

The four-hour procedure duration, with 500 mL blood loss, is consistent with complex adhesiolysis and hernia repair. The 500 mL blood loss (~7% of the estimated 7.2 L blood volume) prompted a precautionary transfusion of 1 unit of PRBC, aligning with guidelines for obese surgical patients where hypovolemia may be underestimated, despite a restrictive Hb threshold of <8 g/dL [18]. Preserving consciousness leads to avoiding postoperative cognitive dysfunction risks in the elderly [19]. The successful outcome, with no intraoperative or postoperative complications and early recovery, supports the efficacy of this anesthetic approach in high-risk patients. Avoidance of sedatives and early ambulation prevents deep vein thrombosis. TSSA-CSE avoided airway instrumentation, reducing pulmonary risks in this obese patient with effusion, and enabled ERAS elements like early mobilization and reduced opioids, as per ERAS guidelines for emergency laparotomy.

The single-patient nature limits generalizability. The expertise for TSSA may not be available in all centers, reducing the technique’s applicability in such centers. Future studies should investigate optimal anesthetic protocols in such high-risk patients and the impact of ketosis on perioperative hemodynamics.

## Conclusions

CSE was safely and successfully used for exploratory laparotomy in an obese 66-year-old female with SBO, incisional hernia, starvation ketosis, and bilateral pleural effusion. The approach provided effective anesthesia and achieved a favorable outcome with enhanced recovery. Hemodynamic stability, avoidance of sedation, and early mobilization can be achieved by careful CSE. These findings highlight the potential of regional anesthesia in high-risk surgical patients. Future studies with larger cohorts and comparative designs are needed to validate the findings and standardize the approach.
